# Einflussfaktoren auf das Einnahmeverhalten von Analgetika bei Patient*innen mit chronischen tumorassoziierten Schmerzen: Eine qualitative Pilotstudie unter Berücksichtigung verschiedener Medikamentengruppen

**DOI:** 10.1007/s00482-023-00765-y

**Published:** 2023-11-13

**Authors:** Marco Richard Zugaj, Andrea Züger, Jens Keßler

**Affiliations:** 1 Universität Heidelberg, Medizinische Fakultät Heidelberg, Klinik für Anästhesiologie, Sektion Schmerzmedizin, Im Neuenheimer Feld 131, 69120 Heidelberg, Deutschland; 2 Nationales Centrum für Tumorerkrankungen (NCT), NCT Heidelberg, eine Partnerschaft zwischen DKFZ und dem Universitätsklinikum Heidelberg, Deutschland, Universität Heidelberg, Medizinische Fakultät Heidelberg, Abteilung für Medizinische Onkologie, Sektion für translationale Medizinethik, Im Neuenheimer Feld 460, 69120 Heidelberg, Deutschland

**Keywords:** Krebs, Nicht-medikamentöse Therapie, WHO-Stufenschema, Multimodale Therapie, Semistrukturiertes Interview, Cancer, Non-drug therapy, WHO staging scheme, Multimodal therapy, Semi-structured interview

## Abstract

**Hintergrund:**

Patient*innen überleben eine Tumorerkrankung durch die Verbesserung der tumorspezifischen Therapie immer länger. Schmerzen sind ein häufiges Symptom. Goldstandard bei tumorassoziierten chronischen Schmerzen ist die multimodale Therapie. Nonadhärenz verursacht hohe Kosten und bringt unter Umständen Patient*innen in Gefahr.

Ziel dieser Studie war es, das Einnahmeverhalten und die subjektive Therapietreue von Patient*innen mit tumorassoziierten chronischen Schmerzen zu untersuchen. Dabei sollte die Perspektive der Patient*innen im Mittelpunkt stehen. Verschiedenen Medikamentengruppen, wie Nicht-Opioid-Analgetika (NOPA), Opioide, Ko-Analgetika und Cannabinoide, aber auch nichtmedikamentöse Verfahren wurden in die Betrachtung eingeschlossen.

**Methode:**

Im Rahmen eines qualitativen Forschungsansatzes wurden semistrukturierte Leitfadeninterviews mit 10 Patient*innen mit chronischen tumorassoziierten Schmerzen durchgeführt. Das gesprochene Wort wurde aufgenommen und transkribiert. Die Auswertung erfolgte im Sinne einer fokussierten inhaltlich strukturierenden Interviewanalyse nach Kuckartz und Rädiker.

**Ergebnisse:**

Es konnten fünf Hauptkategorien definiert werden. Die zentrale Kategorie anhand der Forschungsfrage bildete das „Adhärenzverhalten aus Patient*innensicht“. Den Rahmen der Untersuchung bildete die Kategorie „Medikamentöse Therapie“. Weitere Hauptkategorien waren: „Krankheitsgeschichte“, „Verhältnis zu den Behandelnden“ und „Einstellungen und Überzeugungen“. Insgesamt wurden weitere 77 Unterkategorien gebildet und interpretiert. Das Adhärenzverhalten aus Patient*innensicht unterschied sich zwischen den verschiedenen Medikamentengruppen. Ein palliatives Setting beeinflusste Therapieentscheidungen und Therapieadhärenz. Die angewendeten Medikamentenschemata waren komplex und dynamisch, gerade auch bei mehreren beteiligten Behandelnden. Weiterhin bestand Unklarheit bei der Anwendung von Cannabinoiden. Nichtmedikamentöse Therapien wurden von den Patient*innen marginalisiert. Aus Sicht der befragten Patient*innen waren es weniger die Behandelnden, die Einfluss auf ihr Adhärenzverhalten nahmen, als vielmehr eigene Erfahrungen, Einstellungen und Überzeugungen

**Diskussion:**

Die Untersuchung bezog ergänzend zu bisheriger Literatur in einem qualitativen Setting alle Medikamentengruppen und auch nichtmedikamentöse Therapien gleichgestellt mit ein. Aus der bisherigen Forschung bekannte Adhärenzfaktoren spiegelten sich auch in der subjektiven Wahrnehmung der Gruppe der Patient*innen mit chronifizierten Schmerzen nach Tumorerkrankungen wider. Eine Marginalisierung nichtmedikamentöser Verfahren könnte damit erklärt werden, dass in der Phase einer Chronifizierung multimodale Therapieansätze zu selten konsequent eingesetzt und kontrolliert werden. Medikamentöse und nichtmedikamentöse Therapien sollten daher noch konsequenter auch bei Patient*innen mit tumorassoziierten Schmerzen gleichberechtigt angewendet werden.

**Zusatzmaterial online:**

Die Online-Version dieses Beitrags (10.1007/s00482-023-00765-y) enthält den Interviewleitfaden, den Datenschutzplan und die Liste der Codes.

## Abstract (en)

**Background:**

Patients are surviving tumor diseases longer and longer due to the improvement of tumor-specific therapy and pain is a common symptom. The gold standard for tumor-associated chronic pain is multimodal therapy. Non-adherence causes high costs and may put patients at risk.

The aim of this study was to investigate the adherence behavior and subjective treatment compliance of patients with tumor-associated chronic pain. The focus was on the patients’ perspective. Different groups of medications, such as NOPA, opioids, co-analgesics and cannabinoids, as well as non-drug treatments were included.

**Methods:**

Semistructured guided interviews with 10 patients with chronic tumor pain were conducted within a qualitative research approach. The interviews were recorded and transcribed. The evaluation was using a focused content structuring interview analysis according to Kuckartz and Rädiker.

**Results:**

Five main categories were defined. The central category based on the research question was “Adherence behavior from the patient’s perspective.” The category “Medication therapy” formed the framework of the study. Other main categories were “History of illness”, “Relationship with treatment providers” and “Attitudes and beliefs”. A total of 77 additional subcategories were formed and interpreted. Adherence behavior from the patients’ perspective differed between the different medication groups. A palliative setting influenced treatment decisions and adherence. The medication regimens used were complex and dynamic, especially when there were multiple practitioners involved. Furthermore, there was ambiguity in the use of cannabinoids. Non-drug therapies were marginalized by patients. From the point of view of the patients interviewed, it was not so much the treatment providers who influenced their adherence behavior, but rather their own experiences, attitudes, and convictions.

**Discussion:**

The study included all medication groups and non-drug therapies equally, complementing previous literature in a qualitative setting. Adherence factors known from previous research were reflected in the subjective perception of the group of patients with chronic pain after tumor diseases. Marginalization of non-medication methods could be explained by the fact that multimodal therapy approaches were too rarely constantly used and controlled in the phase of chronification. Therefore, drug and non-drug therapies should be applied even more consistently to patients with tumor-associated pain.

## Hintergrund

Adhärenz bei der Behandlung ist ein wichtiges Bindeglied zwischen Prozess und Ergebnis in der medizinischen Versorgung. Nichtadhärenz verursacht beträchtliche Kosten. Die Nichtberücksichtigung des Grades der Therapieadhärenz kann Auswirkungen auf die Schlussfolgerung aus klinischer Forschung haben [1, 2].

Bei Patient*innen mit einer Tumorerkrankung hat Schmerz als ein die Lebensqualität beeinflussendes Symptom mit 64 % Inzidenz eine sehr hohe Relevanz [3]. Die Chronifizierungsmechanismen bei tumorbedingten Schmerzen gleichen denen bei nicht tumorbedingten Schmerzen [4, 5]. Trotzdem gibt es auch gruppenspezifische Besonderheiten [4–7]. Besondere somatische (Schmerzätiologie, spezifische therapiebedingte Effekte und tumorspezifische Schmerzinduktionsmechanismen), emotionale, kognitive, psychosoziale, verhaltensbezogene, stressbezogene, iatrogene und funktionale Chronifizierungsfaktoren sind bekannt [5]. Patient*innen mit länger anhaltenden tumorbedingten Schmerzen sollten deshalb in einem interdisziplinären Therapiekonzept behandelt werden [5, 8]. Jede Fachgruppe (Ärzt*innen, Pflegende, Psycholog*innen, Physiotherapeut*innen und weitere) bringt dabei ihre Kompetenz in Diagnostik und Therapieverfahren mit ein. Ein zentraler Baustein eines solchen interdisziplinären Konzeptes ist die systemische medikamentöse Therapie mit Opioiden und Nicht-Opioiden nach dem Stufenschema der Weltgesundheitsorganisation (WHO), ergänzt durch Ko-Analgetika und Adjuvantien [9]. Die Auswahl, Kombination und Dosierung der einzelnen Substanzen obliegt dem theoretischen Wissen und der Praxiserfahrung der Behandelnden. Der Erfolg eines medikamentösen Konzepts hängt allerdings nicht nur von dieser fachlichen Expertise ab, sondern auch von der Adhärenz der Patient*innen in Bezug auf Einnahmezeitpunkt, Dosierung und Frequenz der verordneten Medikation [10]. Bei nicht stationär kontrollierter Selbsteinnahme werden nach Rezeptierungen nur etwa 50 % der verschriebenen Arzneimittel tatsächlich eingenommen [11, 12]. Dies verursacht vermeidbare Kosten und gefährdet möglicherweise den Therapieerfolg. Der Forschungsstand zu Medikamentenadhärenz wird im Folgenden zusammengefasst:

### Allgemeine Adhärenzfaktoren bei der medikamentösen Therapie

Britten und Kolleg*innen haben in einer qualitativen Analyse und einer Metaanalyse Faktoren für eine allgemeine Nonadhärenz zur medikamentösen Therapie aufzeigen können [13]. Patient*innen reduzierten eigenmächtig ihre Medikation aufgrund unerwünschter Arzneimittelwirkungen. Auch die ausbleibende Wirkung eines eingenommenen Medikaments konnte zur eigenmächtigen Reduktion oder sogar zum Absetzen des Medikaments führen. Insbesondere bei unklarem kausalem Zusammenhang zwischen Medikament und Nebenwirkung, war das Einnahmeverhalten gefährdet [14]. Fehlte durch ausbleibende Patient*innenedukation schon prinzipiell die Einsicht in die Notwendigkeit der rezeptierten Medikation, konnte es gehäuft zum selbstständigen Absetzen der Medikamente kommen [14, 15]. Eine unzureichende Konkordanz im Sinne einer gestörten Partnerschaft zwischen Behandelnden und Patient*in beeinflusst die Umsetzung des ärztlichen Verordnungsplans [16]. Darüber hinaus hat die grundsätzliche Haltung zur medikamentösen Therapie einen großen Einfluss auf das Einnahmeverhalten [11, 17]. Dowell und Kolleg*innen stellten in einer qualitativen Analyse vier Einflussfaktoren auf das allgemeine Adhärenzverhalten fest: Verständnis, Akzeptanz, Grad der persönlichen Kontrolle und Motivation [18].

### Adhärenzfaktoren bei der medikamentösen Schmerztherapie

Es wird angenommen, dass die Adhärenz bei stark wirksamen Opioiden der WHO-Stufe III besser ist als bei Medikamenten der WHO-Stufe II [19]. Andererseits hat sich im Gegensatz zu amerikanischen Untersuchungen [20] bei einer deutschen Studie gezeigt, dass nichtsteroidale antiinflammatorische Medikamente häufiger eigenmächtig überdosiert werden als Opioide [21]. Die Angst vor Sucht bei der Erstverschreibung von Opioiden kann das Einnahmeverhalten deutlich beeinflussen [9]. Nicht zu vernachlässigen ist die Tatsache, dass Patient*innen mit chronischen Schmerzen und damit verbundenem erhöhtem Leidensdruck und psychischer Anfälligkeit [22, 23] bei der Rezeptierung von Betäubungsmitteln mit geschlechtsspezifischen Häufungen (mehr Männer) [24] einen Übergebrauch entwickeln können [20, 25].

### Adhärenzfaktoren bei tumorassoziiertem chronischem Schmerz

Übersichtsarbeiten fassen folgende Einflussfaktoren zusammen: Durch den Vergleich mit einer fehlenden oder sehr gering dosierten Vormedikation über viele Lebensjahre bei einer nichtmalignen Grunderkrankung kann die Umstellung der Medikation nach der Diagnose von tumorbedingten Schmerzen bedrohlich wirken [9]. Eine enge und regelmäßige Kommunikation mit dem Behandelnden ist bedeutend, gerade bei dynamischer Entwicklung einer Erkrankung und der damit verbundene Notwendigkeit zur Anpassung der Dosierung [26].

Aktuelle qualitative Untersuchungen zur Therapieadhärenz bei tumorassoziierten chronischen Schmerzen fokussieren lediglich die Adhärenz zur Opioidtherapie. Eine qualitative Untersuchung von Seangrung und Kolleg*innen mit 10 Patient*innen mit tumorassoziierten chronischen Schmerzen und Nonadhärenz bei Opioidtherapie zeigte, dass Angst vor den langfristigen Folgen, Opioidnebenwirkungen, unwirksame Schmerzkontrolle, Versuche, die Behandlung akzeptabler zu machen, mangelndes Verständnis und Nichtakzeptanz der Krankheit in Verbindung mit mangelnder Adhärenz standen [27]. Wright und Kolleg*innen hingegen werteten Interviews mit 17 Patient*innen mit tumorassoziierten chronifizierten Schmerzen qualitativ aus. Sie kamen zu dem Ergebnis, dass Veränderungen im Schlaf- und Wachrhythmus bei fortgeschrittener Tumorerkrankung häufig sind und das Einnahmeverhalten in Bezug auf Opioide beeinflussen. Patient*innen verzögerten die Einnahme, ließen Dosierungen aus und wägten zwischen Schmerzen und kognitiven Nebenwirkungen ab [28].

Die meisten Untersuchungen zur Adhärenz nehmen jedoch eine behandlerzentrierte Perspektive ein [29]. Die Patient*innensicht und ihre Deutung auf das Adhärenzverhalten fehlt dabei.

Ziel des vorliegenden Forschungsvorhabens war es daher, die lebensweltlichen Realitäten und subjektiven Wahrnehmungen auf ein verändertes Einnahmeverhalten von medikamentös therapierten Patient*innen mit tumorassoziierten chronischen Schmerzen zu untersuchen. Dabei sollten die Substanzgruppen der medikamentösen und die Methoden der nichtmedikamentösen Schmerztherapie in die Untersuchung einfließen. Im Detail sollte erforscht werden, ob die in der Literatur für eine suboptimale Medikation festgestellten Faktoren sich auch im definierten Studienkollektiv wiederfinden lassen.

## Material und Methoden

Um die Innenperspektive der Patient*innen und deren subjektive Bedeutungszuschreibung bezüglich ihres Adhärenzverhaltens zu ergründen, wurde ein qualitativer Forschungsansatz gewählt.

### Rekrutierung, Einschlusskriterien und Samplingstrategie

Die Patient*innenrekrutierung erfolgte zwischen Mai und Juli 2022. Patient*innen wurden über ein Poster im Wartebereich des Schmerzzentrums des Universitätsklinikums Heidelberg auf die Studie aufmerksam gemacht. Zusätzlich wurden Patient*innen von den behandelnden Schmerztherapeut*innen auf die Studie aufmerksam gemacht.

Das Sampling wurde durch den Studienleiter (MZ) durch eine deduktive (vom theoretischen Vorwissen abhängige) Stichprobenziehung durchgeführt. Um eine möglichst große Varianz und Heterogenität zu erreichen und um die zu generierenden Erkenntnisse in Beziehung zu bestehender Forschungsliteratur zu setzen, sollten, auf der Basis des theoretischen Hintergrunds, die vorab festgelegten Kriterien für die Stichprobenauswahl folgende sein: Patient*innen mit tumorassoziierten chronischen Schmerzen; alle Altersgruppen; beide Geschlechter; bereits angewendetes WHO-Stufenschema I bis III; sämtliche Dynamiken der Schmerzveränderung; gestörtes oder ungestörtes Verhältnis zu den Behandelnden. Die Auswahl basierte auch auf Einträgen in der Patient*innenakte. Das Sample sollte groß genug sein, um eine theoretische Sättigung zu erreichen und um ausreichend viele kontrastierende Fälle zu finden.

Die Einschlusskriterien waren: Patient*innen mit chronischen (mindestens drei Monate bestehend) tumorbedingten Schmerzen (zum Beispiel direktes und verdrängendes Tumorwachstum, Metastasen, Folgen der Tumortherapie) und eine medikamentöse Behandlung der WHO-Stufen I bis III, weil insbesondere die Auswahl der Wirkstoffe das Einnahmeverhalten beeinflussen kann [19].

Ausschlusskriterien waren die fehlende rechtliche Einwilligungsfähigkeit, Alter unter 18 Jahren und unzureichende Deutschkenntnisse.

### Datenerhebung: Qualitative Leitfadeninterviews

Die Erhebungsmethode qualitatives Interview ermöglicht Offenheit, Strukturierung und Spezifizierung zugleich [30]. Der Leitfaden („Interviewleitfaden“ im Online-Zusatzmaterial) wurde auf der Basis von Empfehlungen der Fachliteratur [31], der Forschungsfrage und des Austauschs im Forschungsteam erarbeitet. Der Leitfaden beinhaltet erzählgenerierende Fragen, die Patient*innen anregen sollen, sich zunächst selbst Gedanken um ihr Einnahmeverhalten zu machen. Konkret formulierte Leitfragen, gezieltes Nachfragen und der Einsatz des oben beschriebenen problemorientierten Vorwissens ergänzten die offenen erzählgenerierenden Fragen. MZ (Facharzt für Anästhesie und spezieller Schmerztherapeut, männlich, bisher wenig Erfahrung in qualitativer Forschung, regelmäßige Teilnahme an einer Methodenwerkstadt) führte alle Interviews. Zwischen dem Interviewer und den Studienteilnehmenden bestand keine aktuelle Behandlungsbeziehung. Damit wurde versucht, einer möglichen sozialen Erwünschtheit entgegenzuwirken. Das Vorwissen über die einzelnen Patient*innen beschränkte sich auf die Kategorien der Samplingstrategie.

### Datenaufbereitung

Während des Interviews wurde das gesprochene Wort mit einem digitalen Recorder aufgenommen (Philips DPM6700 Komplettset für Autor und Assistenz, Philips GmbH Market DACH Hamburg, Deutschland) und anschließend von einer Mitarbeiterin der Forschungsgruppe wörtlich transkribiert (Philips Diktier- und Wiedergabe-Software SpeechExec 10, Philips GmbH Market DACH Hamburg, Deutschland). Die Verschriftlichung des gesprochenen Worts wurde konsequent nach Transkriptionsregeln von Kuckartz und Rädiker vorgenommen [32]. Der Datenschutz, die Pseudonymisierung und die Anonymisierung der Rohdaten erfolgte gemäß eines geprüften Datenschutzplans (Datenschutzplan im Online-Zusatzmaterial).

### Datenanalyse

Die textförmig protokollierten Einzelfälle wurden mit der inhaltlich strukturierenden qualitativen Analyse nach Kuckartz und Rädiker [32, 33], konkret in der Umsetzung der fokussierten Interviewanalyse [32], ausgewertet. Die Analyse erfolgte mit Hilfe einer Datenanalysesoftware (MAXQDA Analytics Pro Ausbildung, VERBI Software, Berlin, Deutschland).

Die systematische und fokussierte Inhaltsanalyse erfolgte zwischen Oktober 2022 und März 2023. Sowohl induktive (aus dem Material neu generierte) als auch deduktive (auf dem theoretischen Vorwissen basierende) Ansätze wurden dabei berücksichtigt. Diese Kombination vereint Offenheit und Theoriegeleitetheit und ermöglicht so einen breiten Zugang zum Forschungsinteresse. Ziel sollte eine ganzheitliche Untersuchung lebensweltlicher Phänomene durch Analyse von Einzelfällen sein. Neue Thesen sollten entwickelt werden und Anstoß zu weiterer Forschung geben [33]. Die Auswertung erfolgte dabei in einem vordefinierten sechsstufigen Prozess nach Kuckartz und Rädiker, der in Tab. 1 dargestellt wird.

**Tab. 1:** Detaillierte Darstellung des sechsstufigen Auswertungsprozesses der empirischen Daten nach Kuckartz und Rädiker [32]

Schritt 1: Datenvorbereitung und -exploration	Dieser Schritt beinhaltete das intensive Lesen der Interviews und Schreiben erster Zusammenfassungen und Textmemos
Schritt 2: Deduktive vorläufige Kategorienbildung	Aus dem Leitfaden und dem theoretischen Vorwissen der Untersuchenden wurden erste Kategorien deduktiv gebildet
Schritt 3: Basiscodierung	Mit den vorläufigen Kategorien wurden die Interviews analysiert und Textabschnitte wurden den Kategorien zugeteilt (codiert). Dabei wurden weitere Kategorien definiert und letztendlich in einem festen Kategoriensystem verankert
Schritt 4: Feincodierung	Eine tabellarische Auflistung aller Textpassagen einer Kategorie erlaubte eine tiefergehende Analyse und Ausdifferenzierung in Unterkategorien. Zur weiteren Akzentuierung wurden für ausgewählte Textpassagen Zusammenfassungen geschrieben
Schritt 5: Datenanalyse	Für die Forschungsfrage interessante Daten wurden ausgesucht und kontrastierend gegenübergestellt
Schritt 6: Dokumentation	Kontrolle der Dokumentation aller Einzelschritte. Verfassen eines Forschungsberichts. Archivierung der Daten gemäß Datenschutzkonzept und arbeitsrechtlicher Beratung der zuständigen Ethikkommission

Es wurden verschiedene Techniken angewendet, um die Glaubwürdigkeit der Datenanalyse zu erhöhen. Die Autoren MZ und AZ (promovierte Kulturwissenschaftlerin, weiblich, mehrjährige Erfahrung in qualitativer Sozialforschung) werteten die Interviews 4, 8 und 9 zunächst sukzessive unabhängig voneinander aus und konsentierten danach Schritt für Schritt die Auswertung. Die Codierung erfolgte dabei nach schriftlich fixierten Codierregeln [32]. Gütekriterien für Kategorien wurden definiert und konsequent angewendet [32]. Die Ausdifferenzierung des Kategoriensystems erfolgte bei den induktiv ausgewählten Kategorien „Medikation“, „Kontrolle“ und „Angst“ ebenfalls im interdisziplinären Diskurs (Kulturwissenschaften – AZ und Anästhesie/Schmerztherapie – MZ). Ein kleinschrittiger Audit-Trail wurde angelegt, sodass Rezensent*innen einzelne Phasen der Studienplanung, Datenakquise und Datenauswertung verfolgen konnten. Die Erstellung des Forschungsberichts erfolgte nach den „Standards for Reporting Qualitative Research (SRQR)“-Richtlinien [34].

### Ethikvotum

Gegen die Durchführung der Studie wurden nach berufsrechtlicher Beratung durch die Ethikkommission der Medizinischen Fakultät Heidelberg unter der internen Nummer S‑099/2022 keine Bedenken geäußert. Die Studie wurde im Deutschen Register Klinischer Studien unter DRKS00028517 registriert und nach der aktuellen Fassung der Deklaration von Helsinki durchgeführt. Alle Studienteilnehmende erteilten schriftlich ihre Einwilligung.

## Ergebnisse

10 Patient*innen mit chronischen tumorassoziierten Schmerzen wurden in die Studie eingeschlossen. Es nahmen sieben Frauen und drei Männer teil. Das Alter der Teilnehmer*innen lag zwischen 20 und 90 Jahren. Die Erstdiagnose der Tumorerkrankung lag zwischen einem und 17 Jahren zurück. Eine Übersicht gibt Tab. 2.

**Tab. 2:** Zusammenfassung der Patient*innencharakteristika und besonderer Interviewbedingungen

Interview	Geschlecht	Alter (Jahre)	Tumordiagnose	Besondere Bedingungen	Schmerzcharakter und Auswahl an Begleiterkrankungen
1	Weiblich	60–70	2005	Eine Begleitperson. Nicht störungsfreies Verhältnis zu den vielen Behandelnden Hohe Pregabalin-Dosis bei gleichzeitig chronischer Niereninsuffizienz	Vorbestehend: Lumbale Bandscheibenschäden mit Radikulopathie Chronisches Schmerzsyndrom mit psychischen und somatischen Faktoren Restless-Legs-Syndrom Diabetes mellitus Typ 2 Gemischte sensomotorische diabetische Polyneuropathie Adipositas Chronische Niereninsuffizienz Ulcus cruris rechts bei Stauungsdermatose
2	Männlich	50–60	2021	Eine Begleitperson Schwierige Kommunikation nach Unterkiefer-Operation	Neuropathische Schmerzen nach Unterkieferresektion und Radiatio bei Plattenepithelkarzinom
3	Weiblich	70–80	2017	Eine Begleitperson Patientin leidet an beginnender Demenz, ist aber selbstversorgend	Chemotherapieinduzierte Polyneuropathie
4	Weiblich	50–60	2020	Cannabinoidtherapie	Schmerzen durch ossäre Metastasierung Vorbestehend: Multiple Sklerose
5	Weiblich	60–70	2017	Rezidivtumor	Chemotherapieinduzierte Polyneuropathie Ulzerierendes Tumorwachstum Vorbestehend: Migräne ohne Aura
6	Männlich	70–80	2010	Eine Begleitperson	50 Zyklen Immuntherapie mit Pembrolizumab seit 2015 Studientherapie mit Avelumab/Cemiplimab seit 2020, hierunter onkologische Befundkonstanz
7	Weiblich	60–70	2019	Einfache Sprache	Neu aufgetreten: Schmerzexazerbation durch ossäre Metastasierung
8	Weiblich	20–30	2011	Gutes Verhältnis zu den Behandelnden Hohe Resilienz	Neu aufgetreten: Metastasen bedingen stärkste Neuralgien an Plexus brachialis
9	Weiblich	60–70	2015	Patientin verweigert die Einnahme von Nicht-Opioid-Analgetika	Neu aufgetreten: Metastasen bedingen stärkste Neuralgien an Plexus lumbalis. Z. n. frustraner Computertomographie-gesteuerter Alkoholneurolyse
10	Männlich	80–90	2018	Eine Begleitperson Positive Lebenseinstellung	Neu aufgetreten: Pathologische Wirbelkörperfraktur bei ossärer Metastasierung

**Abb. 1:**
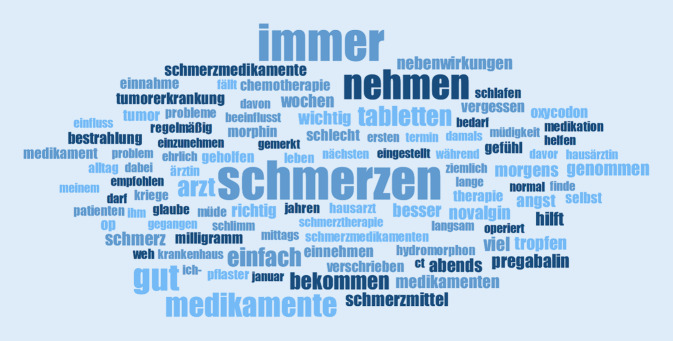
Wortwolke mit den am häufigsten durch die Teilnehmer*innen genannten Wörtern. Pronomen, Füllwörter und Artikel wurden herausgefiltert, da kein Sinnzusammenhang zur Forschungsfrage durch sie entsteht

Über 5 h Interviewmaterial wurde transkribiert und ausgewertet. Eine Wortwolke (Abb. 1) gibt einen Überblick über häufig verwendete Wörter der Teilnehmer*innen. Pronomina, Artikel und Füllwörter wurden hierbei herausgefiltert. „Schmerz“ „Tabletten“ „immer“ „Medikamente nehmen“ kamen häufig vor. Aber auch „gut“ und „helfen“ wurden häufig genannt.

**Abb. 2:**
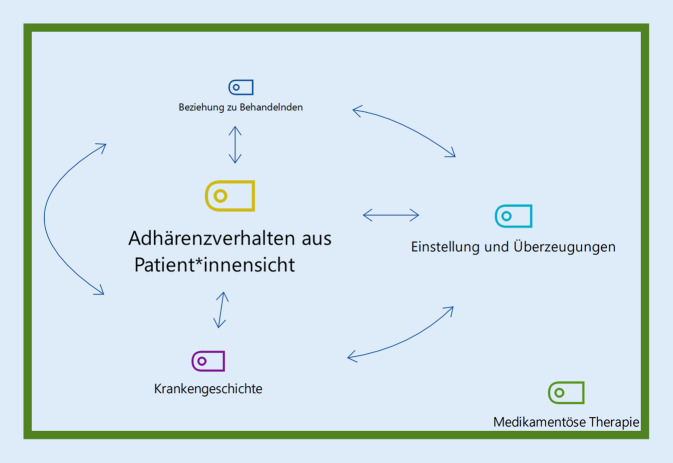
Beziehungsanordnung der fünf Hauptkategorien. „Adhärenzverhalten aus Patient*innensicht“ bildet die Basis der Forschungsfrage (*Gelb*, groß). „Medikamentöse Therapie“ gibt den Themenrahmen vor (*Grün*, Rahmen). Die Hauptkategorien „Beziehung zu Behandelnden“ (*Dunkelblau,* klein), „Einstellung und Überzeugungen“ (*Hellblau*) und „Krankengeschichte“ (*Lila*) beeinflussen sich gegenseitig

Die Basis aus der Forschungsfrage heraus bildete die Kategorie „Adhärenzverhalten aus Patient*innensicht“. Den thematischen Rahmen gab die Kategorie „Medikamentöse Therapie“ vor (Grüner Rahmen). Als weitere Hauptkategorien konnten „Einstellungen und Überzeugungen“, „Krankengeschichte“ und „Beziehung zu Behandelnden“ herausgearbeitet werden. Die Hauptkategorien beeinflussten sich gegenseitig und es gab teilweise thematische Überschneidungen. Eine Einordnung der Beziehung der Hauptkategorien untereinander gibt Abb. 2. Die Hauptkategorien konnten weiter in Unterkategorien der Grade 1 bis 3 ausdifferenziert werden. Es wurden insgesamt 82 Kategorien definiert („Liste der Codes“ im Online-Zusatzmaterial) und 770 Segmente codiert.

### Adhärenzverhalten aus Patient*innensicht

Alle Teilnehmenden gaben an, dass sie ihre Medikamente in der Regel wie verschrieben einnähmen. Im Laufe des Gesprächs wurden jedoch bei acht Patient*innen im Detail Abweichungen vom Therapieplan festgestellt. Unterkategorien des „Adhärenzverhaltens aus Patient*innensicht“ bei der Medikamenteneinnahme waren die „unbewusste Therapieänderung“ wie das „Vergessen der Einnahme“ und „versehentliche Änderung“ (hier meistens versehentliche Fehler beim Vorrichten der Medikamente) sowie „bewusste Therapieänderung“, hier insbesondere die „Einnahme nach Bedarf, statt nach Schema“, und das „Variieren des Einnahmeintervalls“. Eine Patientin antwortete auf die Frage, ob sie ihre Schmerzmedikamente schon mal vergessen habe:

„P: Nein. (…) (I Und‑) Es kann passieren, dass ich es mal etwas später merke. Gerade die abends kann es schon mal passieren, dass ich mal die etwas später nehme als um acht, dann mal um neun, weil ich halt abgelenkt war durch den Fernseher und dann erst an meine Tabletten gedacht habe. Aber ganz vergessen tue ich die nie.“

### Medikamentöse Therapie

Die Forschungsfrage zielt insbesondere auf das Verhältnis zu verschiedenen Medikamentengruppen ab. Somit ist die Kategorie „Medikamentöse Therapie“ ein Bezugsrahmen, der die anderen Kategorien einschließt. Explizit soll der Fokus nicht alleine auf Opioiden liegen, sondern alle Medikamentengruppen sollen in die Betrachtung einbezogen werden.

#### Vergleich von Medikamentengruppen

##### Opioide.

Die Patient*innen berichteten, dass ein sofortiges Einsetzen einer Wirkung wie bei starken Opioiden und unretardierter Galenik die Adhärenz erhöhe:

„P: Das habe ich bei der ganze Überlegung jetzt grad vergessen diese Pflaster. Ja, die haben natürlich ich sage mal sehr geholfen. Die restlichen Medikamente habe ich dann total vergessen, ja.“

Ein Patient berichtete über einen Fehlgebrauch von Opioiden und Benzodiazepinen im Rahmen einer palliativen Versorgung. Nach einer neuartigen Antikörpertherapie und hierunter onkologischer Befundstabilisierung über viele Jahre, musste ein Entzug erfolgen:

„P: Also ganz leicht, es ging ja letztendlich da drum dieser Mann lebt nicht mehr lange und der kriegt alles, was er benötigt und das wird auch – das wurde wirklich großzügig gehandhabt. Muss ich ganz ehrlich sagen. Nur die Hausärztin mussten natürlich dann immer unterschreiben dafür. Das ist mir klar. Und dann war die Hausärztin mal im Urlaub, dann war der andere Arzt und der hat gesagt: ‚Stopp da stimmt etwas nicht. Sie können nicht so viel Tavor und Morphin (unv.) nehmen, das geht einfach nicht, ja.‘“

##### Gabapentinoide.

Auch wenn bei einem Auslassen einer Dosis eine Verschlechterung des Allgemeinbefindens auftritt, erhöhte dies die Bereitschaft, die Medikation regelhaft einzunehmen. Dies wurde bei Opioiden und Gabapentinoiden beschrieben:

„P: Also mir ging es so schlecht, dass ich überhaupt nichts gegessen habe, weil keine Medikamente genommen und dann habe ich gemerkt, dass ich – ich habe zuerst nicht kapiert, woher kommt diese Unruhe? Und jetzt weiß ich inzwischen, dass es davon kommt, weil ich Methadon Tropfen nicht genommen habe und auch von diese Pregabalin nicht genommen habe.*“*

##### Nicht-Opioid-Analgetika (NOPA).

Die Medikamentenwirkung nicht zu spüren, zum Beispiel bei NOPA, erschwerte die Adhärenz. NOPA wurden in den substanzgruppenspezifischen Nebenwirkungsprofilen nicht unterschieden:

„P: Wenn ich sie nicht brauche, dann nehme ich sie nicht. Weil ich auch nicht das Novalgin immer trinke, nicht? Wenn ich keine Schmerzen habe, warum soll ich das nehmen?“

„I: Novalgin eine Allergie und Penicillin eine Allergie und Paracetamol-“

„P: Vertrage ich, aber bringt mich nicht weiter.“

##### Antidepressiva.

Eine Patientin berichtete über die Wirkung von Antidepressiva auf ihre Stimmung. Von einer analgetischen Wirkung von Antidepressiva wurde nicht berichtet:

„P: Ich bin eine alleinerziehende Mutter. Die waren die Gedanken, die immer gekommen sind und ich war sehr traurig immer deshalb hat mir diese Mirtazapin-Tabletten geholfen.“

##### Cannabinoide.

Drei Patient*innen sprachen über Cannabinoide. Grenzen zwischen Cannabinoiden als Medikation und zum hedonistischen Gebrauch verschwammen dabei. Die beruhigende Wirkung, nicht die Analgesie, war subjektiv am gewinnbringendsten:

„P: Ja, also wie gesagt ich habe es geraucht gehabt und die Beschaffung ja (.) irgendwie ist man drangekommen, jetzt ja. Und man muss natürlich aufpassen, weil man wusste immer nicht genau wieviel das jetzt ist.“

„P: Also ich konnte super drauf schlafen, was auch unheimlich wichtig war. Mein Schlaf war schon immer sehr gestört dadurch. Ich konnte zum ersten Mal wieder richtig durchschlafen. Und mir ging es dann einfach den nächsten Tag einfach nur gut. Das war so eine Erleichterung ganz komisch.“

#### Nichtmedikamentöse Therapie

Sieben Patient*innen berichteten von einer nichtmedikamentösen Tumorschmerztherapie, überwiegend von Physiotherapie. Keine Patient*in konnte subjektiv einen bedeutenden Effekt auf den tumorassoziierten Schmerz erkennen:

„P: Also dieses Physiotherapiegedöns – Entschuldigung, dass ich das so sage – nicht schlecht. Es ist gut. Aber für die Schmerzen finde ich, also wenn man so Schmerzen hat wie ich (..), bringt es eigentlich nicht viel.“

Über die Bedeutung psychologischer Verfahren bei tumorassoziierten Schmerzen berichtete lediglich eine Patientin:

„P: Es ist natürlich auch wichtig, dass die Psyche quasi stabil ist. Das macht auch für mich viel aus, ob ich viel Schmerz empfinde oder nicht, aber die Medikamente machen auf jeden Fall schon den größten Teil aus.“

### Beziehung zu Behandelnden

Patient*innen mit chronischen Schmerzen nach Tumorerkrankungen haben insgesamt selten über die Stakeholder des Gesundheitssystems gesprochen. Einige Patient*innen berichteten über negative Erfahrungen im Sinne einer paternalistischen Kommunikation durch die Behandelnden (Subkategorie „Kommunikation der Behandelnden“):

„P: Ja, es wird nicht nachgefragt: ‚Warum wird das jetzt so gemacht?‘ oder ‚Wieso ist das so?‘ Das ist einfach so.“

Teilnehmende gaben an, kaum durch den einzelnen Arzt oder die einzelne Ärztin in ihrem Adhärenzverhalten beeinflusst zu sein. Dafür gaben die Patient*innen an, von der Institution Krankenhaus beeinflusst zu werden. Therapien von anderen Disziplinen, die für die Patient*innen subjektiv wichtig waren, wie zum Beispiel die onkologische Tumortherapie, färbten auf das Adhärenzverhalten zur Schmerztherapie ab. Wir nannten diesen Effekt „Halo-Effekt“, da das Behandlungssetting auf die Therapieadhärenz aller beteiligten Behandlungsdisziplinen ausstrahlte (Subkategorie „Behandlungssetting“):

„P: Ja, für mich schon eher die Umgebung. Also es ist – ja weiß jetzt gar nicht, ob ich habe jetzt noch nie so wirklich negative Erfahrungen mit einem Arzt gemacht habe.“

Keine Diagnose zu haben, zu wenig Informationen zu erhalten, oder „Watchful Waiting“ wurde von den Teilnehmenden als Bürde empfunden (Subkategorie „Behandlungsfortschritt“):

„P: Und der guckt die Wunde und sagt es ist alles in Ordnung. Und sage ich, aber sage ich nicht in Ordnung. Merke hier einen Knochen. Und habe Kopfschmerzen. (..) Wo das normal das wieder geht weg, aber ich sage nichts geht weg, nichts.“

### Einstellung und Überzeugungen

Die Adhärenz zu einer medikamentösen Schmerztherapie hing subjektiv am meisten von der inneren Einstellung und den Überzeugungen der Patient*innen ab. Unterkategorien von „Einstellungen und Überzeugungen“ waren: „Verständnis“, „Akzeptanz“, „Motivation“ und „Angst“.

#### Angst

Ein häufig genannter Aspekt der Angst war es, einer Schmerzexazerbation hilflos ausgesetzt zu sein:

„P: Aber ich kann ihnen etwas über die schlimmsten Schmerzen (.) erzählen, die ich hatte in der Zeit. Also da waren sie echt soweit, da hätte ich, wenn mir jemand eine Pistole hingelegt hätte und gesagt hätte: ‚Hier, das ist deine Hilfe.‘ Dann hätte ich mich wahrscheinlich erschossen. So schlimm waren die Schmerzen.“

Vor Opioiden als Substanzgruppe hatten vier Patient*innen explizit keine Angst. Auch die Angst vor einer möglichen Abhängigkeit wurde selten erwähnt, wenn auch Entzugssymptome bei Opioiden und bei Gabapentinoiden häufig berichtet wurden. Sieben Patient*innen gaben Angst vor unspezifischen Nebenwirkungen an. Einigen Patient*innen war die Einnahme von NOPA besonders unangenehm. Verstärkt wurde das Unbehagen durch hohe Tablettenanzahl und nicht merkbare Medikamentenwirkung bei NOPA. Vor allem um Leber und Niere machten sich Patient*innen Sorgen, weil diese Organe vom Abbau der eingenommenen Medikamente besonders betroffen seien:

„P: Meine Angst wieder, dass meine Leber und meine Niere dann irgendwann mal vielleicht etwas kommt, bei dem dann einfach diese ganze Geschichte vom Körper nicht mehr so abbaubar ist.*“*

Thorax- oder Geschlechtsorgane wurden nicht als gefährdet erwähnt. Über zentralnervöse und gastrointestinale Nebenwirkungen konnten alle Patient*innen aus eigener Erfahrung berichten. Eine Unterkategorie war die Angst vor der „Endgültigkeit einer Medikation“ bei chronischer Erkrankung:

„P: Nein, (..) also meine Angst ist, ich später muss das immer nehmen. Das ich will ich nicht.“

#### Akzeptanz

Die Kategorie „Akzeptanz der medikamentösen Therapie“ überschnitt sich bei acht Patient*innen mit der Kategorie „suffiziente medikamentöse Therapie“:

„I: Und das Hydromorphon, da sagen sie da haben sie eine ganz klare Wirkung und deshalb nehmen sie das dann auch mit gutem Gewissen (P Ja. Ja) Oder sozusagen nehmen es auch ein, auch wenn es ein bisschen zweischneidig ist (P Ja) und bei dem Novalgin, da haben sie eben die Wirkung nicht gemerkt (P Ja) und sagen dann, dann ist es mir nicht wert so viele Tabletten einzunehmen, wenn ich es dann eh nicht so richtig merke. (P Ja, genau)“

Bei drei Patient*innen wurde auch die Kategorie „unerwünschte Nebenwirkungen“ mit „Akzeptanz der medikamentösen Therapie“ gemeinsam erwähnt. Die drei Patient*innen fielen gleichzeitig durch außergewöhnliche Resilienz auf:

„P: Ja, wieso soll ich da Angst haben? Die helfen mir. Wissen sie, ich bin ein Optimist und ich muss kämpfen und ich muss das machen, dass ich für meine liebe Frau und für die Kinder und die Enkelkinder da bin. […] Wenn er gleich immer denkt: ‚Oh und das und das.‘ Und dann kriegt er – dann werden die Organe schlechter.“

#### Motivation

Am häufigsten wurde als Motivation für eine Adhärenz zur verordneten Schmerztherapie die Hoffnung auf Schmerzlinderung genannt. Oft mit der Intention, den Alltag weiterhin bewältigen zu können:

„P: Eine, eine auf jeden Fall mehr Lebensqualität, oder zumindest so weit schmerzfrei zu werden, dass ich wieder am sozialen Leben teilnehmen kann, dass ich nicht immer Angst haben muss, dass mir jetzt das Bein weggeht.“

Ein palliatives Setting hat die Motivation zu Adhärenz erhöht und zu aggressiverer Therapie geführt:

„P2: Aber, aber die davor war auch die die Dings noch da gewesen [Frau] die Ärztin, die war nämlich von der Palliativ – Und die hat das auch immer verschrieben gehabt. Die hat gesagt: ‚Wenn sie Schmerzen haben, dann können sie die ruhig nehmen. Sie müssen nicht unter Schmerzen leiden.‘ So hieß das. Und er hat dann halt immer gedacht: ‚Ich habe Schmerzen. Jetzt nehme ich das.‘ Bis irgendwann mal der Punkt kam, an dem wir gemerkt haben, er nimmt nicht so viel, wie sein sollte.*“*

#### Verständnis

Häufig genannt und von besonderer Bedeutung für das Verständnis der Schmerzmedikation waren eigenes Erfahrungswissen und das Erfahrungswissen von Peers. Dies führte zu Annahmen und Vermutungen über Wirkung und Nebenwirkungen und deren Relation bei der Schmerzmedikation. Nebensächlich für die Überzeugung war dabei, ob es sich um korrektes medizinisches Wissen handelte. Verständnis für Erkrankung und Medikation konnte selbst erlangt (Beipackzettel), oder durch Aufklärung durch Stakeholder des Gesundheitssystems erzeugt werden:

„P: Also mein Arzt hat immer gesagt: ‚So viel Pregabalin, wie sie nehmen – sie müssten den ganzen Tag schlafen.‘ Und ich habe eine Zeit lang gehabt, in der ich gesagt habe: ‚Ne, brauche ich nicht. Im Gegenteil, ich bin fit wie ein Turnschuh (.)‘“

#### Kontrollmechanismen der Einnahme

Es wurden verschiedene Kontrollmechanismen beschrieben, um die Adhärenz eigenständig zu steigern: Am häufigsten wurde die Hilfe durch Bezugspersonen angegeben. Grundsätzlich musste die Applikationstechnik der Medikation für die Patient*innen umsetzbar sein (Tropfen – Tabletten). Patient*innen gaben an, einen zentralen Ansprechpartner für die Medikation und einen einheitlichen aktuellen Medikamentenplan zu haben erleichtere die regelmäßige Einnahme. Ebenso feste Einnahmerituale, um keine Einnahme zu vergessen:

„P: Ich mache das automatisch, weil ich ja weiß ich nehme die zusammen mit den Medikamenten, die ich auch für meine Tumorbehandlung brauche.“

Die Tabletten über eine Woche vorzurichten erleichtere die regelmäßige Einnahme, jedoch mit dem Nachteil, wenn die Medikation umgestellt werde, müssen die vorgerichteten Tabletten entsorgt werden:

„P: Und das (.) das geht mir jedes Mal so. (.) Das ging mir die letzte seitdem wie lange war ich jetzt in der [Lungenfachklinik]? Sechs, vier Wochen. Seit den letzten vier Wochen, in denen ich grundsätzlich einmal in der Woche den Mülleimer aufmache und die Dinger da reinwerfe.“

### Krankengeschichte

Die anderen Hauptkategorien wurden von der Kategorie „Krankengeschichte“ beeinflusst. „Medikamentenanamnese“, „Demografische Daten“, „Behandlungsverlauf“, „Krankheitsverlauf“ und „Schmerzverlauf“ waren Teil der individuellen Krankengeschichte.

„I: Das ging immer höher, immer höher. Und das Pflaster wurde dann auch durch eine Tablette ersetzt, auch von auch mit einem-“

„P: Auch mit – mit Oxycodon wurde das ersetzt.“

„I: Mit Oxycodon und dann ging es erstmal weiter und dann war es ja eine lange Zeit. Dann sind wir ja immer noch vor 17 Jahren so zu sagen.“

„P: Ja, ich sag ja das geht mit diesem Schmerzmitteln Oxycodon, hauptsächlich Oxycodon, das Pregabalin kam erst ziemlich spät, oder?“

## Diskussion

Ziel dieser Studie war es, das Einnahmeverhalten und die subjektive Therapietreue von Patient*innen mit chronischen tumorassoziierten Schmerzen zu untersuchen. Ein Schwerpunkt wurde insbesondere auf die Unterscheidung verschiedener Medikamentengruppen des WHO-Stufenschemas gelegt. Ein qualitativer Studienansatz im Sinne einer inhaltlich strukturierenden Interviewanalyse wurde gewählt. Die Analyse der empirischen Daten erbrachte fünf Hauptkategorien, die sich untereinander jeweils beeinflussten und teilweise überschnitten. Im Mittelpunkt stand die Zielkategorie „Adhärenzverhalten aus Patient*innensicht“, den thematischen Rahmen der Befragung bildete die Kategorie „Medikamentöse Therapie“. Der Themenrahmen umfasste alle Medikamentengruppen und zusätzlich in der Offenheit der Befragung auch nichtmedikamentöse Therapien und unterschied sich daher von vorhandener qualitativer Literatur.

Beeinflussungsfaktoren konnten unter „Beziehung zum Behandelnden“, „Einstellungen und Überzeugungen“ und „Krankengeschichte“ subsumiert werden. Alle Hauptkategorien konnten in Unterkategorien weiter ausdifferenziert werden, sodass letztendlich Faktoren erarbeitet werden konnten, die die umfangreiche Literatur zu allgemeinen Adhärenzverhalten um eine Kontrastierung und Akzentuierung im Falle der Patient*innengruppe mit chronischen tumorassoziierten Schmerzen ergänzten.

Es konnte ein heterogenes Sample mit unterschiedlichem Alter, Schmerzentitäten, Schmerzintensitäten und unterschiedlicher Schmerzdauer ausgewählt werden. In einem inkludierenden Ansatz wurde auch eine Patientin mit beginnender Demenz eingeschlossen, deren subjektive Wahrnehmung evaluiert und in die Auswertung integriert. Begleitpersonen führten ebenfalls nicht zum Ausschluss, da in der lebensweltlichen Realität unserer Patient*innen eben diese psychosoziale Faktoren von Bedeutung sind. Gerade hier ist der Vorteil einer qualitativen Methode gegenüber einer quantitativen Methode zu sehen.

Insgesamt geht aus den Ergebnissen der empirischen Datenanalyse hervor, dass Medikamente mit einer weniger unmittelbaren Wirkung weniger plangetreu eingenommen werden. Bezogen auf Opioide ist dieser Effekt bekannt und auch bei Patient*innen mit tumorassoziierten Schmerzen von Enting und Kollegen beschrieben [19]. Im vorgestellten Kollektiv wurden jedoch nicht nur WHO-Stufe-II-Medikamente weniger strikt eingenommen, sondern vor allem WHO-Stufe-I-Medikamente. Ergänzend zu den Untersuchungen von Wright [28] und Seangrung [27], konnten wir feststellen, dass bei NOPA eine Differenzierung anhand substanzgruppenspezifischer Nebenwirkungen durch die Patient*innen oft nicht erfolgte [9]. Waren die Medikation suffizient und die Nebenwirkungen gering, förderte dies die Adhärenz.

Wirz und Kollegen beschrieben 2016 Chronifizierungsmechanismen und Abhängigkeitspotenziale bei tumorassoziiertem Schmerz [5]. In unserem Kollektiv berichtete ein Patient von einem nach onkologischem Therapieerfolg notwendigen Entzug bei Opioid- und Benzodiazepinfehlgebrauch. Unsere Patient*innen erwähnten zwar eine generelle Angst vor Abhängigkeit bei Opioiden und auch bei Gabapentinoiden, diese war jedoch nicht entscheidend für die Therapieadhärenz. Das Gefühl einer ärztlichen Supervision der Therapie konnte diese Angst lindern. Generell wurde berichtet, dass unter dem Gesichtspunkt der Palliation weniger Widerstände gegen eine aggressivere Therapie auftraten. Insbesondere durch die regelhafte Verwendung von schnellwirksamen Opioiden unterschied sich die Gruppe der Patient*innen mit tumorassoziierten Schmerzen von anderen chronifizierten Schmerzerkrankten.

Patient*innen berichteten von Stigmatisierung bei der Cannabinoidtherapie. Ein Verschwimmen von hedonistischem Gebrauch und medikamentöser Cannabinoidtherapie war erkennbar. Indikation von und Therapie mit Cannabinoiden schienen bei Patient*innen und auch Behandelnden weiter unklar. Hier könnte weitere Forschung notwendig sein, um Subgruppen von Patient*innen mit positiver Kosten-Nutzen-Relation zu identifizieren und Try-and-Error-Ansätze bei der Cannabinoidtherapie in Zukunft zu vermeiden [35, 36].

Neben den medikamentösen Behandlungsmethoden gibt es zahlreiche nichtmedikamentöse Behandlungsverfahren, die in der Tumorschmerztherapie Anwendung finden. Dazu zählen unter anderem Strahlentherapie, transkutane elektrische Nervenstimulation (TENS), psychosomatische Therapieansätze, Verhaltens- und Bewegungstherapie, physikalische Therapieansätze, Akupunktur und operative Verfahren. Als nichtmedikamentöse Therapie wurde in unserem Sample die Physiotherapie erwähnt und diese wurde als insuffizient beschrieben, entgegen wissenschaftlicher Erkenntnisse [37]. Es könnte sein, dass zu wenig schmerzspezifische Physiotherapie durchgeführt oder eine konsequente Anwendung autonomer Übungen nicht kontrolliert wurde. Die psychische Verfassung war subjektiv nur für eine Patientin von Bedeutung bei der Schmerzwahrnehmung und Verarbeitung. Es könnte sein, dass in unserem Sample keine oder zu wenige schmerzspezifische psychotherapeutische Maßnahmen angewendet wurden [37]. Abhilfe könnten hier unter Umständen digitale Gesundheitsanwendungen (DiGa) schaffen, deren Effektivität bereits bestätigt wurde [38]. Stand Herbst 2023 gibt es jedoch weiterhin keine DiGa, die für die Anwendung bei chronischen tumorassoziierten Schmerzen indiziert ist [39]. Aussagen über Adhärenzfaktoren zu einer nichtmedikamentösen Therapie können auf Basis der Interviews nicht getroffen werden. In unserem Sample wurde keine interdisziplinäre, multimodale, stationäre oder teilstationäre Schmerztherapie (IMST) durchgeführt, jedoch eine ambulante Therapie mit Behandelnden verschiedener Disziplinen (Ärzt*innen, Psycholog*innen, Physiotherapeut*innen), die durch die Schmerztherapeut*innen orchestriert wurde.

Nebenwirkungen und Grenzen der Schmerzreduktion zu akzeptieren erforderte von den interviewten Patient*innen eine besondere Resilienz (Aufrechterhaltung einer positiven Anpassung durch Einzelpersonen trotz erheblicher Widrigkeiten [40]).

Eine einfache Therapie konnte die Adhärenz erhöhen. Nicht förderlich war eine komplexe Therapie mit dynamischem Wechsel des Therapieschemas [26] durch mehrere Behandelnde. Wright et al. beschrieben bereits eine Grenze bei drei Medikamenten für eine höhere Adhärenz [28].

Weiterhin wurde bereits beschrieben, dass eine fehlende Konkordanz zwischen Behandelnden und Patient*innen subjektiv zu einer Beeinflussung der Adhärenz führen kann [14]. In unserem Studiensample wurde jedoch weniger die direkte Beziehung zum Behandelnden als das übergeordnete Setting der Therapie als wichtiger Einflussfaktor auf die Adhärenz wahrgenommen. Dabei hat zum Beispiel die Adhärenz zur onkologischen Therapie auch zu einer Adhärenz zur schmerztherapeutischen Therapie geführt.

Zusammenfassend konnten wir somit zeigen, dass die Gruppe der Patient*innen mit tumorassoziierten chronischen Schmerzen Besonderheiten aufweist. Dabei vereint sie Charakteristika der Gruppe der Patient*innen mit tumorassoziierten Schmerzen und der Gruppe der Patient*innen mit chronischen nicht tumorassoziierten Schmerzen. Es fällt auf, dass diese Zwischenstellung es mitunter schwierig macht, die untersuchte Gruppe einzuordnen. Dies führt auch dazu, dass sie regelmäßig aus Forschungsarbeiten zum chronischen Schmerz ausgeschlossen wird [38].

### Limitationen

- Generelle Einschränkungen zur Verallgemeinerung unserer Ergebnisse: In der Regel sind die Fallzahlen in der qualitativen Forschung klein und das Sampling entspricht nicht den Kriterien einer Zufallsauswahl. Deshalb kann qualitative Forschung anders als die mit großen Fallzahlen arbeitende quantitative Forschung keine Generalisierung in Form von statistischer Repräsentativität beanspruchen [32]. Die Generalisierung geschieht in diesen Fällen allerdings in Form einer empirisch begründeten Theorie oder als Erkennen von Mustern und nicht in Form der Ermittlung statistischer Signifikanz [32]. Wir haben deshalb umfänglich über die Samplegröße und die Auswahl der Patient*innen im Methodenteil Auskunft gegeben.
- Durch das Setting der Studie in den Räumen des Universitätsklinikums und die Person des Interviewführenden kann keine Fremdheitsannahme erfolgen. Anpassungen der Aussagen der Teilnehmenden im Sinne sozialer Erwünschtheit können nicht komplett ausgeschlossen werden. Interviewbasierte Analysen können generell einen falsch hohen Eindruck der Adhärenz durch ein Verschweigen von Nonadhärenz aus Scham oder sonstigen Beweggründen erzeugen [1].
- Die Interviews waren durch grundsätzliche Offenheit und Nichtwerten geprägt. Der Erzählfluss wurde nicht gestoppt. Das Interview unterschied sich also grundsätzlich von der Struktur eines Anamnesegesprächs. Trotzdem könnte das Vorwissen des Interviewenden über Patient*innen und Konzepte der Adhärenz die Interviewführung beeinflusst haben.

## Fazit für die Praxis

- Bei Patient*innen mit chronischen tumorassoziierten Schmerzen liegen besondere Bedingungen für ein Adhärenzverhalten zur medikamentösen Therapie vor. Diese können im Patient*innengespräch verbalisiert werden.
- Charakteristika von Patient*innen mit nicht-tumorbedingten chronischen Schmerzen und von Patient*innen mit tumorassoziierten Schmerzen werden vereint.
- Trotz der diskutierten Besonderheiten der Gruppe der Patient*innen mit chronischen tumorassoziierten Schmerzen sollten sie nicht aus Forschungsarbeiten zu chronischen Schmerzen ausgeschlossen werden.
- Erkenntnisse der multimodalen Therapie mit medikamentösen und nichtmedikamentösen Elementen sollten auch bei Patient*innen mit tumorassoziierten chronischen Schmerzen angewendet werden.

## Supplementary Information

Interviewleitfaden

Datenschutzplan

Liste der Codes

## Abkürzungen

DiGa: Digitale Gesundheitsanwendung
I: Interviewende Person
NOPA: Nicht-Opioid-Analgetika
P: Befragte Person
P2: Begleitperson
SRQR: Standards for Reporting Qualitative Research
WHO: World Health Organisation
