# Supplementary material for: Factors influencing analgesic use patterns in patients with chronic tumor-associated pain. A qualitative pilot study considering different groups of medications
Source: Schmerz. 2023 Nov 13;38(6):422–32. [Article in German] doi: 10.1007/s00482-023-00765-y (PMC11576800; doi:10.1007/s00482-023-00765-y)
Supplement: Supplementary file 1 — Interviewleitfaden [file 482_2023_765_MOESM1_ESM.pdf]

Leitfaden: Welche Faktoren beeinflussen das Einnahmeverhalten von Schmerzmedikamenten gemäß einem ärztlichen Verordnungsplan bei Palliativpatienten mit tumorbedingten Schmerzen?

I. Gesprächseröffnung: Allgemeiner Rahmen		
**Inhaltliche Aspekte**	**Aufrechterhaltungsfragen**	**Nachfragen**
Vorstellung der eigenen Person<br><br>Ideen und Ziele des Interviews<br><br>Technische Angaben zum Interview (Tonaufnahme, Dauer ca. 30min.)<br><br>Vorstellung des Patienten (Name, Geburtsdatum, Art der Tumorerkrankung mit Datum der Erstdiagnose)<br><br>Datum/Zeitraum des ersten tumorbedingten Schmerzerlebens		

II. Hauptteil – Frühes Stadium des Interviews

Wie war Ihr Schmerzerleben im Verlauf der Tumorerkrankung?

Wie würden Sie Ihre Erfahrung mit der Einnahme von Schmerzmedikamenten beschreiben?

Was ist ihre aktuelle Schmerzmedikation?

Wie fühlen Sie sich, wenn Sie aktuell Schmerzmedikamente einnehmen?

Haben Sie schon einmal vergessen, Schmerzmedikamente einzunehmen?

Nehmen Sie die Schmerzmedikamente immer genauso, wie der Arzt sie Ihnen verschrieben hat?

Inhaltliche Aspekte	Aufrechterhaltungsfragen	Nachfragen
Subjektive Bedeutungszuschreibung<br><br>Wichtigkeit/Relevanz der verschriebenen Medikation	Fällt Ihnen dazu sonst noch was ein?<br><br>Gibt es sonst noch was?<br><br>Und dann?<br><br>Und sonst?<br><br>Was meinen Sie damit?	• Passiert das Vergessen der Einnahme von Schmerzmedikamenten in der Hektik<br>• Was trägt aus ihrer Sicht sonst dazu bei, dass Schmerzmedikamente nicht wie geplant eingesetzt werden?<br>• Spielt die Person des verschreibenden Arztes aus Ihrer Sicht eine Rolle für ihr Einnahmeverhalten

III. Hauptteil – Spätes Stadium des Interviews

Erzählen Sie doch mal: Wie managen Sie Ihre tägliche Medikation?

Welche Nebenwirkungen von Schmerzmedikamenten haben sie schon gehabt?

Wurden Ihr Alltag und ggf. auch Ihr Berufsleben von Nebenwirkungen verändert?

Was bedeutet es für Ihren Alltag, regelmäßig Schmerzmedikamente einnehmen zu müssen?

Was denken Sie über die Balance zwischen der häufigen Einnahme von Schmerzmedikamenten und ihrem Alltag?

Was bedeutet es für sie, täglich Schmerzmedikamente wie ärztlich verordnet, einnehmen zu müssen?

Haben Sie schon einmal Schmerzmedikamente willentlich über oder unterdosiert?

Haben Sie Bedenken/Angst vor morphinähnlichen Substanzen?

Was ist die Folge dieser Bedenken/Angst?

Inhaltliche Aspekte	Aufrechterhaltungs-fragen	Nachfragen
Ablauf bei neu aufgetretenen oder exazerbierten Schmerzen<br><br>Erleben von Ärzten während der Rezeptierung<br><br>Hilfreiche/belastende Elemente	Fällt Ihnen dazu sonst noch was ein?<br><br>Gibt es sonst noch was?<br><br>Und dann?<br><br>Und sonst?<br><br>Was meinen Sie damit?	• Wie viele und welche Schmerzmittel müssen Sie momentan einnehmen?<br>• Gibt es spezielle Medikamente, bei denen es Ihnen widerstrebt, sie einzunehmen?<br>• Ist das Verhältnis zum verschreibenden Arzt dadurch merklich gestört?<br>• Gibt es Schmerzmedikamente, die sie eher als andere eigenmächtig unter- oder überdosieren würden?

IV. Der Abschluss des Interviews

Wenn Sie nun zum Schluss unseres Interviews einmal selber in einer Reihenfolge von 1-3 sagen müssen, welche drei Dinge Ihr Einnahmeverhalten von Schmerzmedikamenten am meisten beeinflusst hat, was wären diese?

Inhaltliche Aspekte	Aufrechterhaltungsfragen	Nachfragen
	Fällt Ihnen dazu sonst noch was ein?<br>Gibt es sonst noch was?<br>Und sonst?<br>Was meinen Sie damit?	• Was würden Sie denn nach einem Rollentausch als Arzt gegenüber einem Schmerzpatienten anders machen?<br>• Was wünschen Sie sich für Ihre zukünftige Schmerztherapie?

Offene Nachfrage zum Abschluss

Von meiner Seite aus sind wir langsam ans Ende des Interviews angelangt. Gibt es noch etwas, das wichtig für Sie ist, hier in unserem Interview aber noch nicht zur Sprache gekommen ist?

Herzlichen Dank für Ihre Teilnahme! Ich wünsche Ihnen alles Gute.
