# Supplementary material for: Factors influencing analgesic use patterns in patients with chronic tumor-associated pain. A qualitative pilot study considering different groups of medications
Source: Schmerz. 2023 Nov 13;38(6):422–32. [Article in German] doi: 10.1007/s00482-023-00765-y (PMC11576800; doi:10.1007/s00482-023-00765-y)
Supplement: Supplementary file 2 — Datenschutzplan [file 482_2023_765_MOESM2_ESM.pdf]

Datenschutzplan der Studie nach Mayermann und Porzelt

Quelle: Mayermann A, P.M. (2014). Hinweise zur Anonymisierung von qualitativen Daten. Forschungsdatenbildung informiert. https://www.forschungsdaten-bildung.de/files/fdb-informiert-nr-1.pdf. Zugegriffen: 15. September 2023

Geplantes praktisches Vorgehen zur Anonymisierung<br>1. Es wird bei Interviews bereits vor deren Durchführung der Patient/ die Patientin darauf hingewiesen werden, dass er/sie im Interview keine Namen oder identifizierenden Merkmale (z. B. Ortsangaben) nennen, die ihn/sie selbst oder Dritte betreffen.<br>2. Die Anonymisierung wird bereits bei der Transkription durchgeführt bzw. sensible Informationen bereits hier für eine spätere Anonymisierung gekennzeichnet (z. B. durch die Verwendung von eckigen Klammern). Um einschätzen zu können, welche Informationen sensibel sind und welche nicht, wird empfohlen, das Material vorher vollständig anzuhören.<br>3. Es wird ein Anonymisierungsprotokoll angefertigt, in dem alle Ersetzungen, Zusammenfassungen (Aggregationen) und Löschungen dokumentiert sind, und es sollte festgehalten werden, welche Personen die Anonymisierung durchgeführt haben. Das Anonymisierungsprotokoll wird von den anderen Daten getrennt aufbewahrt.<br>4. Die Rohdaten werden nach Auswertung, spätestens aber nach drei Jahren gelöscht.<br>5. Es ist keine Folgeauswertung der erhobenen Daten geplant. Eine Parallele Auswertung der Daten durch ein Mitglied einer anderen Fachrichtung kann durchgeführt werden, um eine sichere interindividuell konsistente Interpretation zu gewährleisten. Die auszuwertenden transkribierten Daten sind dann bereits anonymisiert.<br><br>Da selbst bei einer faktischen Anonymisierung – wie sie gesetzlich vorgeschrieben ist (Personen sind so zu anonymisieren, dass sie nur mit einem „unverhältnismäßig hohen Aufwand“ reidentifiziert werden könnten) - das Restrisiko einer Reidentifikation nicht auszuschließen ist, wird zusätzlich zur Anonymisierung auch eine Zugriffskontrolle implementiert werden, um die Schutzrechte der Untersuchungspersonen zu gewährleisten.
