# Supplementary material for: Factors influencing analgesic use patterns in patients with chronic tumor-associated pain. A qualitative pilot study considering different groups of medications
Source: Schmerz. 2023 Nov 13;38(6):422–32. [Article in German] doi: 10.1007/s00482-023-00765-y (PMC11576800; doi:10.1007/s00482-023-00765-y)
Supplement: Supplementary file 3 — Liste der Codes [file 482_2023_765_MOESM3_ESM.pdf]

Liste der Codes

Liste der Codes	Häufigkeit
Codesystem	770
Adhärenzverhalten aus Patient*innensicht	15
Adhärenzverhalten aus Patient*innensicht\Unbewusste Therapieänderung	0
Adhärenzverhalten aus Patient*innensicht\Unbewusste Therapieänderung\Vergessen der Einnahme	9
Adhärenzverhalten aus Patient*innensicht\Unbewusste Therapieänderung\Aus Versehen	1
Adhärenzverhalten aus Patient*innensicht\Bewusste Therapieänderung	11
Adhärenzverhalten aus Patient*innensicht\Bewusste Therapieänderung\Variieren des Einnahmeintervalls	4
Adhärenzverhalten aus Patient*innensicht\Bewusste Therapieänderung\Einnahme nach Bedarf statt nach Schema	3
Beziehung zu Behandelnden	0
Beziehung zu Behandelnden\Behandlung	0
Beziehung zu Behandelnden\Behandlung\Eigene Entscheidung treffen	2
Beziehung zu Behandelnden\Behandlung\unzufrieden mit watchfull waiting	3
Beziehung zu Behandelnden\Behandlung\unzufrieden ohne Diagnose	2
Beziehung zu Behandelnden\Kommunikation	0
Beziehung zu Behandelnden\Kommunikation\paternalistische Kommunikation	4
Beziehung zu Behandelnden\Kommunikation\unzufrieden mit mangelnder Kommunikation	1
Beziehung zu Behandelnden\Kommunikation\zufrieden mit Erklärungen	5
Beziehung zu Behandelnden\Kommunikation\Einfache Wortwahl	2
Beziehung zu Behandelnden\Kommunikation\Gehört fühlen	1
Beziehung zu Behandelnden\Setting	3

Beziehung zu Behandelnden\Setting\"Halo-Effekt"	3
Beziehung zu Behandelnden\Setting\Vertrauen	4
Einstellung und Überzeugungen	0
Einstellung und Überzeugungen\Angst	1
Einstellung und Überzeugungen\Angst\Angst vor Schmerzexacerbation	2
Einstellung und Überzeugungen\Angst\Opioide Abhängigkeit	1
Einstellung und Überzeugungen\Angst\Wechselwirkung mit anderen Erkrankungen und Medikamenten	1
Einstellung und Überzeugungen\Angst\Angst vor unklaren Nebenwirkungen	7
Einstellung und Überzeugungen\Angst\Keine Angst (vor Opioiden)	5
Einstellung und Überzeugungen\Angst\Angst um Organe (siehe Memo 127)	7
Einstellung und Überzeugungen\Angst\Angst vor Entgültigkeit	2
Einstellung und Überzeugungen\Akzeptanz	0
Einstellung und Überzeugungen\Akzeptanz\Nicht-Akzeptieren der Erkrankung	0
Einstellung und Überzeugungen\Akzeptanz\Akzeptieren der Erkrankung	3
Einstellung und Überzeugungen\Akzeptanz\Nichtakzeptieren der medikamentösen Therapie	5
Einstellung und Überzeugungen\Akzeptanz\Akzeptieren der medikamentösen Therapie	24
Einstellung und Überzeugungen\Motivation	0
Einstellung und Überzeugungen\Motivation\Reduktion der Medikation	1
Einstellung und Überzeugungen\Motivation\Palliatives Setting	5
Einstellung und Überzeugungen\Motivation\Kosteneffizienz	2
Einstellung und Überzeugungen\Motivation\Hoffnung auf Schmerzlinderung	9
Einstellung und Überzeugungen\Motivation\Hoffnung auf Schmerzlinderung\Alltag bewältigen	9
Einstellung und Überzeugungen\Verständnis	10

Einstellung und Überzeugungen\Verständnis\eigenes Erfahrungswissen	7
Einstellung und Überzeugungen\Verständnis\eigenes Erfahrungswissen\Erfahrung von Peers	1
Einstellung und Überzeugungen\Verständnis\Aufklärung durch Shareholder Gesundheit	11
Einstellung und Überzeugungen\Verständnis\Aufklärung durch Shareholder Gesundheit\Kenntnis des Medikationsplans	2
Einstellung und Überzeugungen\Verständnis\Aufklärung durch Shareholder Gesundheit\Kenntnis des Medikationsplans\Dosisangabe	10
Einstellung und Überzeugungen\Verständnis\eigeninitiierte Verständis	5
Einstellung und Überzeugungen\Verständnis\Annahmen und Vermutungen	10
Einstellung und Überzeugungen\Verständnis\Annahmen und Vermutungen\Kausalitäten	1
Einstellung und Überzeugungen\Verständnis\Annahmen und Vermutungen\Korrektes medizinisches Wissen	5
Medikamentöse Therapie	15
Medikamentöse Therapie\Kontrollemechanismen	0
Medikamentöse Therapie\Kontrollemechanismen\Hilfe durch Bezugsperson	10
Medikamentöse Therapie\Kontrollemechanismen\Einnahmerituale	10
Medikamentöse Therapie\Kontrollemechanismen\Ein zentraler Ansprechpartner	5
Medikamentöse Therapie\Kontrollemechanismen\Einheitlicher Medikamentenplan	2
Medikamentöse Therapie\Kontrollemechanismen\Tabletten vorrichten	6
Medikamentöse Therapie\Kontrollemechanismen\Aplikationstechnik umsetzbar?	5
Medikamentöse Therapie\Multimodale Therapie	8
Medikamentöse Therapie\Sucht oder Kontrollverlust	6
Medikamentöse Therapie\Vergleich von Medikamentengruppen	22
Medikamentöse Therapie\Tablettenanzahl	9
Medikamentöse Therapie\Wirkung der Medikamente	1

Medikamentöse Therapie\Wirkung der Medikamente\suffizient	26
Medikamentöse Therapie\Wirkung der Medikamente\insuffizient	11
Medikamentöse Therapie\Wirkung der Medikamente\Nebenwirkungen	3
Medikamentöse Therapie\Wirkung der Medikamente\Nebenwirkungen\unerwünschte Nebenwirkungen	38
Medikamentöse Therapie\Wirkung der Medikamente\Nebenwirkungen\gewünschte Nebenwirkungen	2
Medikamentöse Therapie\Wirkung der Medikamente\Auslassen von Medikamenten	6
Medikamentöse Therapie\Wirkung der Medikamente\Auslassen von Medikamenten\End of dose failure	3
Krankengeschichte	0
Krankengeschichte\Medikamentenanamnese	12
Krankengeschichte\Demographische Daten	10
Krankengeschichte\Behandlungsverlauf	48
Krankengeschichte\Krankheitsverlauf	34
Krankengeschichte\Schmerzverlauf	49
Zitierfähige Stellen	23
Blumen am Wegesrand	7
Wünsche für die Zukunft	7
Sonstiges	14
GELB	3
ROT	69
BLAU	63
VIOLETT	24
